# A generic optimization and learning framework for Parkinson disease via speech and handwritten records

**DOI:** 10.1007/s12652-022-04342-6

**Published:** 2022-08-26

**Authors:** Nada R. Yousif, Hossam Magdy Balaha, Amira Y. Haikal, Eman M. El-Gendy

**Affiliations:** Computer and Control Systems Engineering Department, Faculty of Engineering, Mansoura University, Mansoura, Egypt

**Keywords:** Feature extraction, Hyperparameters optimization, Machine learning (ML), Parkinson disease (PD), Speech segmentation, Transfer learning (TL), Voice segmentation

## Abstract

Parkinson’s disease (PD) is a neurodegenerative disorder with slow progression whose symptoms can be identified at late stages. Early diagnosis and treatment of PD can help to relieve the symptoms and delay progression. However, this is very challenging due to the similarities between the symptoms of PD and other diseases. The current study proposes a generic framework for the diagnosis of PD using handwritten images and (or) speech signals. For the handwriting images, 8 pre-trained convolutional neural networks (CNN) via transfer learning tuned by Aquila Optimizer were trained on the NewHandPD dataset to diagnose PD. For the speech signals, features from the MDVR-KCL dataset are extracted numerically using 16 feature extraction algorithms and fed to 4 different machine learning algorithms tuned by Grid Search algorithm, and graphically using 5 different techniques and fed to the 8 pretrained CNN structures. The authors propose a new technique in extracting the features from the voice dataset based on the segmentation of variable speech-signal-segment-durations, i.e., the use of different durations in the segmentation phase. Using the proposed technique, 5 datasets with 281 numerical features are generated. Results from different experiments are collected and recorded. For the NewHandPD dataset, the best-reported metric is 99.75% using the VGG19 structure. For the MDVR-KCL dataset, the best-reported metrics are 99.94% using the KNN and SVM ML algorithms and the combined numerical features; and 100% using the combined the mel-specgram graphical features and VGG19 structure. These results are better than other state-of-the-art researches.

## Introduction

Parkinson’s disease (PD) is a chronic neurological disorder resulting from the diminishment in the levels of dopamine as a result of a shortage of dopamine-producing cells in the brain. As the brain is the control center of the entire human body, any deficiency in the work of its cells affects the signals propagating to the different parts and causes different symptoms. In the case of PD, symptoms can be classified into motors and non-motors (Politis et al. 2010). In the first category, patients suffer from symptoms including (1) tremors, (2) Freezing of Gait (FoG), (3) muscle rigidity, (4) Fear of Falling (FoF), (5) slow movements, (6) impaired posture, (7) micrographia, and (8) voice abnormality (Berus et al. 2019). In the second category, symptoms include (1) depression, (2) dementia, (3) sleep disorders, (4) anxiety, (5) slow thinking, and (6) fatigue (Almeida et al. 2019).

PD mostly affects people after 60 years old. However, it sometimes affects patients in the 40s because of genetic reasons (De Lau and Breteler 2006). PD can affect both genders, but it has been proven that male patients are affected more compared to females (Lamba et al. 2021). The main concern with PD is that symptoms appear clearly after the loss of about $80\%$ of the dopaminergic cells (Sveinbjornsdottir 2016). Till this moment, researchers are unable to specify the reason behind that disease. There is no treatment for PD until the recent moment, but symptoms can be controlled by proper medications (Hireš et al. 2021). Therefore, early detection of PD can help patients have a self-sufficient life (Gupta et al. 2018).

Several diagnostic markers of PD can be used, of which the handwriting and voice signals because of their low cost and less time consumption compared to MRI or other brain tests are selected. Patients with PD have problems in motor skills including those used for writing due to the effect of muscle rigidity, shacking, and slow movement (Dias et al. 2020). Although changes in handwriting are hardly perceivable in the early stages of the disease, it is still an essential biomarker of PD diagnosis (Kamran et al. 2021). With the evolution in deep learning, visual features can be extracted automatically and used to train a network of several layers to correctly classify patients of PD from normal people.

Abnormalities in voice signals are another biomarker of the existence of PD. Patients with PD have their voice turned softer, with fast and monotonous speech. These abnormalities might be unnoticeable to normal people and need experts (Ali et al. 2019). Speech impairments can be observed from either running speech or continuous vowel phonation (Rizvi et al. 2020). Diagnosing PD from voice changes has become very popular in recent research due to its simplicity and time-saving. Therefore, the use of voice tests in the diagnoses of PD is of trending interest (Caliskan et al. 2017).

The application of deep learning (DL) to image classification has guaranteed better accuracy (Xiao et al. 2021b, 2021a; Balaha et al. 2021b). Convolutional neural network (CNN) is the commonly used DL approach in the field of medical imaging because of their robustness in automatic feature extraction (Li et al. 2021; Balaha et al. 2021a; Huynh et al. 2016). Applications of CNN in medical imaging include pancreas segmentation (Roth et al. 2015), brain tumor segmentation (Havaei et al. 2017; Guttman et al. 2003), liver cancer segmentation Li et al. 2018), detection of cerebral microbleeds (Dou et al. 2016), COVID-19 (Balaha et al. 2021e, 2021d; Bahgat et al. 2021), skin cancer detection Połap 2019), and Alzheimer’s disease (Helaly et al. 2021; Khagi et al. 2018). To build a CNN from scratch, big data must be available to train the network efficiently. However, in case of limited available data, it is preferable to use existing models that were previously trained such as ImageNet, and “transfer” all the knowledge in the model targeted to be trained on the new data. This approach is called transfer learning (TL) (Cao et al. 2013; Balaha et al. 2021c).

The use of metaheuristic algorithms (i.e., optimizers) in solving optimization problems is currently the most common approach (Sörensen and Glover 2013). With their flexibility, an optimal solution can be achieved (Yousri et al. 2021). Unfortunately, concerning the no free lunch (NFL) theorem, a single optimization algorithm can outperform other algorithms in some problems, but it can also have bad performance for other problems. Therefore, new algorithms are continuously being built. Examples of the currently available algorithms include Genetic Algorithms (Holland 1992), Particle Swarm Optimization (Kennedy and Eberhart 1995), Bat Algorithm (Yang and Gandomi 2012), Red Fox Optimization Algorithm (Połap and Woźniak 2021), and Marine Predators Algorithm (Faramarzi et al. 2020). The use of metaheuristic algorithms in learning the hyperparameters of CNN is of great interest in recent researches (Singh et al. 2021; Loussaief and Abdelkrim 2018; Wang et al. 2019; Khalid and Javaid 2020; Soon et al. 2018).

Machine learning (ML) algorithms are usually used in data classification problems (Aggarwal et al. 2021; Raheja et al. 2021; Thapliyal et al. 2021; Chakradar et al. 2021). The most important step of ML is to successfully extract the essential features that guarantee robust classification. Different ML algorithms are available such as decision trees (Rokach and Maimon 2005) and support vector machines (Steinwart and Christmann 2008). However, they all have approximately the same principle; i.e., the machine is trained on data for correct classification (Jordan and Mitchell 2015).

In the current study, a comprehensive generic framework for early and accurate detection of PD using both handwritten images and speech signals is proposed. It consists of four phases, namely (1) datasets collection, (2) pre-processing, (3) hyperparameters optimization, and (4) classification, to handle both data types. For handwritten images, patients are required to draw specific shapes. The resulting shapes are then diagnosed by the system. Here, 8 pre-trained CNN models via TL, namely (1) ResNet50 (He et al. 2016), (2) VGG16, (3) VGG19 (Simonyan and Zisserman 2014), (4) MobileNet (Howard et al. 2017), (5) MobileNetV2 (Sandler et al. 2018), (6) MobileNetV3Small, (7) MobileNetV3Large (Howard et al. 2019), and (8) InceptionResNetV2 (Szegedy et al. 2017) are used. To optimize the hyperparameters, an optimization algorithm called Aquila Optimizer (AO) (Abualigah et al. 2021) is utilized. This algorithm is based on the behavior of Aquilas during the hunting process. Due to the limitation of available handwriting data, different data augmentation techniques are applied to increase the diversity dataset to avoid overfitting.

For the speech signals, 16 numerical feature extraction, 5 graphical feature extraction, and 4 machine learning (ML) algorithms are used. The ML algorithms are (1) Decision Tree (DT) (Loh 2011), (2) Support Vector Machine (SVM) (Vapnik 2013), (3) Naïve Bayes (NB) Tsangaratos and Ilia 2016), and (4) K-Nearest Neighbor (KNN) (Zhang et al. 2017). A new approach in the features’ dataset preparation concerning the speech signals’ segmentation is proposed. It involves segmenting the voice signals into segments of different durations and combining them into a heterogeneous dataset. 5 heterogeneous datasets with 281 numerical features each and 5 graphical features are generated. To optimize the hyperparameters, the grid search (GS) (LaValle et al. 2004) is used with the ML algorithms and AO with the pre-trained CNN models.

### Contributions

The contributions of the presented work can be summarized in the following points:

- Proposing a generic framework for early and accurate diagnosis of PD using a combination of disorders in both handwritten images and speech signals.
- Using a combination of disorders in both the handwritten and speech signals.
- Using 8 pre-trained CNN models via TL to classify PD using the handwritten images and 4 ML algorithms to diagnose PD from speech signals.
- Applying 16 numerical feature extraction and 5 graphical feature extraction algorithms that generated 281 numerical features and 5 graphical features.
- Optimizing the CNN and ML hyperparameters using GS and AO.
- Proposing a new approach in voice segmentation using different durations to increase the diversity and heterogeneity features.

### Paper organization

The rest of the paper is divided into 4 sections. Section 2 presents some state-of-the-art studies about the diagnosis of PD. Section 3 describes the methodology used to build the proposed framework. The experimental results, discussion, and comparative study of the proposed framework are discussed in Sect. 4. Section 5 presents the current study limitations. Section 6 presents the conclusions and future works.

## Results

A lot of research has been done to diagnose PD using intelligent techniques. Pereira et al. (2015) made a dataset called HandPD of 55 subjects with 37 PD and 18 healthy subjects. They applied different ML classifiers, i.e., NB, SVM, and optimum-path forest (OPF), on the extracted features. They reported a maximum accuracy of 78.9% using the NB classifier. In their other trial, Pereira et al. (2016b) performed many experiments using CNN. They applied different train/test split ratios and different image resolutions. They could achieve an accuracy of 80.19%.

In their next study, Pereira et al. (2016a) applied different metaheuristic techniques, namely firey algorithm, bat algorithm, and molecule swarm optimization, to extract features from the handwriting dataset. They used CNN in classification. They could achieve an accuracy of 90.385%. In Pereira et al. (2018), Pereira et al. applied CNN to the same dataset and could achieve an accuracy of 95%. Senatore et al. (2019) applied cartesian genetic programming (CGP) for the classification of PD. The authors used the HandPD dataset, and from their results, they could achieve a global accuracy of 72.36%.

PaHaW dataset was also used in many studies. For example, Impedovo (2019) applied an SVM classifier with a linear kernel on it. They could achieve an accuracy of 98.44%. Naseer et al. (2020) used AlexNet architecture via TL in the diagnosis of PD. They applied different augmentation techniques to increase the dataset size and could achieve an accuracy of 98.28%. Kamran et al. (2021) applied different CNN structures via TL on a combination of different datasets, namely HandPD, NewHandPD, and Parkinson’s Drawing datasets. They also applied different augmentation techniques. They could report a maximum accuracy of 99.22% using the AlexNet structure.

Several studies using speech data are also made. For instance, Caliskan et al. (2017) used two speech datasets, namely the Oxford Parkinson’s Disease Detection (OPD) dataset and Parkinson Speech Dataset with Multiple Types of Sound Recordings (PSD). They applied a deep neural network classifier for the detection of PD. They could achieve an average accuracy of 93.79% using the OPD dataset. Sakar et al. (2013) collected voice samples from 20 subjects to create a PD voice dataset. After extracting the essential features, they used SVM and KNN classifiers. They could report a maximum accuracy of 77.5% using the SVM classifier.

Zahid et al. (2020) used AlexNet structure via TL to learn acoustic features and generate spectrograms. They used the pc-Gita dataset and could achieve an accuracy of 99.7%. Tuncer and Dogan (2019) proposed a novel pre-processing technique called the octopus-based pooling technique. They also applied Singular Value Decomposition for feature extraction and Neighborhood Component Analysis for feature selection. They could report a maximum accuracy of 97.62% using the 1-Nearest Neighbor classifier.

These studies are just examples of many other studies (Parziale et al. 2021; Qasim et al. 2021; Orozco-Arroyave et al. 2016; Tsanas et al. 2012; Kurt et al. 2019; Solana-Lavalle et al. 2020; Kurt et al. 2018; Kuresan et al. 2021). The application of IoT has also guaranteed better management and control Sun et al. 2021; Bhardwaj et al. 2021; Połap 2018).

### Summarization

Table 1 summarizes the discussed related studies.

**Table 1:** Related studies summarization concerning PD

Reference	Year	Approach	Dataset	Dataset type	Pros.	Cons.	Best accuracy
Pereira et al. Pereira et al. 2015)	2015	ML using NB, SVM, and OPF	HandPD	Image	Proposing “HandPD” dataset	Achieved accuracy is low	78.9% using NB
Pereira et al. Pereira et al. 2016b)	2016	CNN			Proposing an extension to the “HandPD” dataset using signals from a smartpen from meander and spiral drawings	(1) The use of an imbalanced dataset with more healthy samples and (2) the usage of tablet-based devices requires specific conditions for good quality	80.19%
Pereira et al. Pereira et al. 2016a)	2016	Metaheuristics + CNN			Usage of metaheuristic algorithms to tune the hyperparameters	The usage of imbalanced dataset with more healthy samples	90.39%
Pereira et al. Pereira et al. 2018)	2018	CNN			(1) CNN is applied for learning features from handwritten dynamics and (2) proposing “NewHandPD” dataset extracted by the use of a smartpen	Process of the time-series data in a black-box manner	95%
Senatore et al. Senatore et al. 2019)	2019	CGP			The usage of Cartesian Genetic Programming to provide explicit classification rules	Poor results for spiral images	72.36%
Impedovo et al. Impedovo 2019)	2019	SVM with a linear kernel	PaHaW		Usage of velocity signals	Useful in online handwriting only	98.44%
Naseer et al. Naseer et al. 2020)	2020	CNN using AlexNet			(1) The usage of fine-tuned pretrained models and (2) the usage of k-fold cross-validation	(1) No consideration of dimensionality reduction and (2) vulnerability to acoustic conditions	98.28%
Kamran et al. Kamran et al. 2021)	2021	CNN using AlexNet, GoogLeNet, VGG, and ResNet	HandPD, NewHandPD, and Parkinson’s Drawing datasets		(1) The usage of several datasets and (2) high achieved accuracy.	Poor accuracy in case of scratch CNN	99.22% using AlexNet
Sakar et al. Sakar et al. 2013)	2013	SVM, KNN	Speech data	Voice	Proposal of voice dataset for Parkinson’s disease	Results are biased	77.5%
Caliskan et al. Caliskan et al. 2017)	2017	DNN	OPD and PSD		Remote diagnosis ability	Low accuracy	93.79%
Tuncer et al. Tuncer and Dogan 2019)	2019	SVM, 1NN, DT, and logistic regression	Vowel		Gender classification is taken into account	The usage of small data	97.62% by 1NN
Zahid et al. Zahid et al. 2020)	2020	AlexNet	pc-Gita		(1) The usage of deep features of speech and (2) proving that pronunciation of vowels are sufficient in diagnosis	Poor accuracy for isolated words	99.7%

## Methodology

**Fig. 1:**
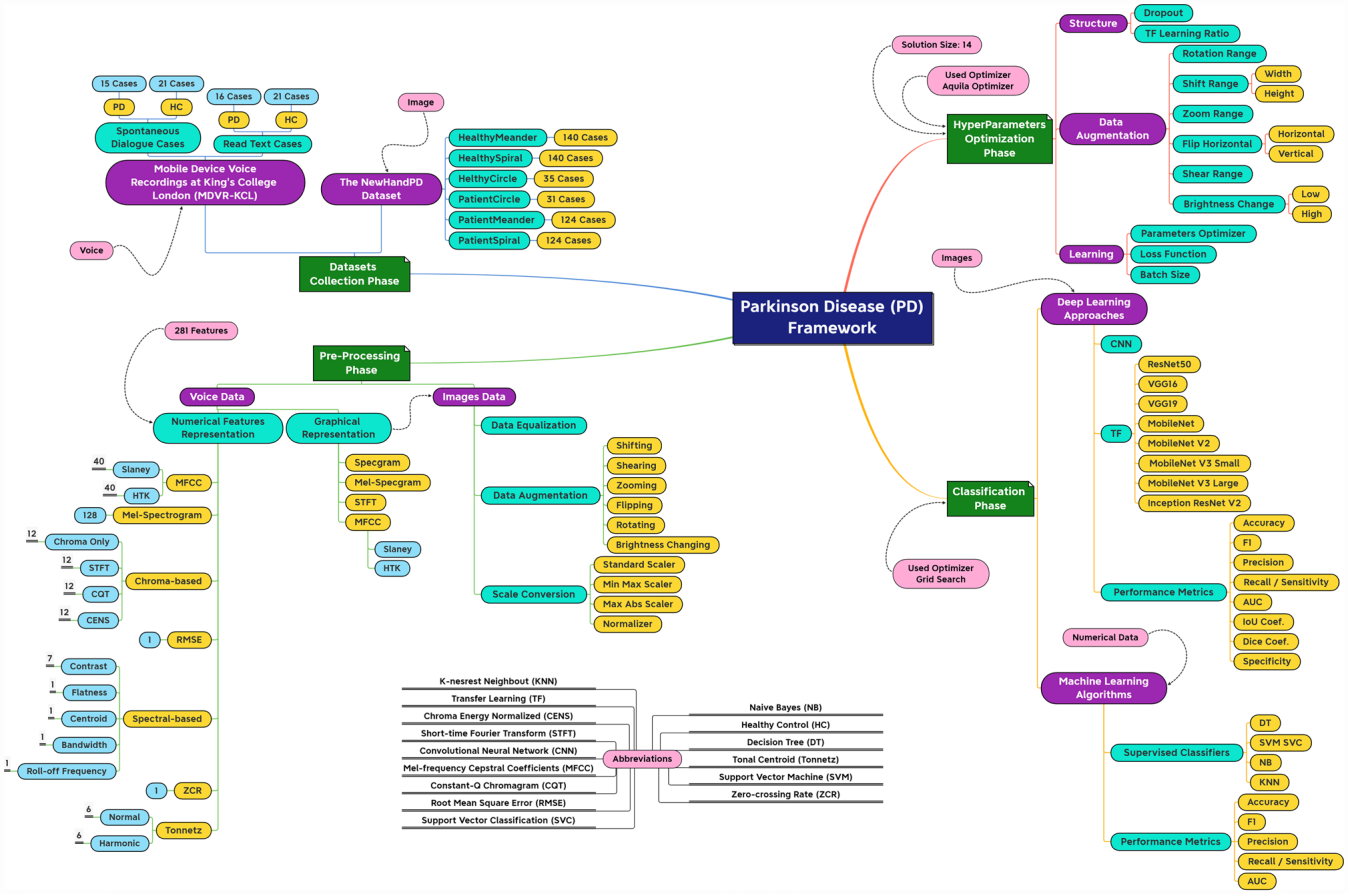
The Parkinson diseases learning and optimization framework

The current study suggests a generic framework (shown in Fig. 1) for Parkinson’s disease learning and optimization. The framework is divided into four major phases. They are (1) datasets collection, (2) pre-processing, (3) classification, and (4) hyperparameters optimization phases. In summary, the data collection phase presents insights into the used Parkinson datasets. The pre-processing phase is responsible for handling the images (and voice) data and preparing them for the classification phase. The classification and hyperparameters optimization phases focus on optimizing a pre-trained CNN model and handling numerical records using ML algorithms. The phases are discussed in the following subsections.

### Datasets collection phase

The current study works on two public datasets. They are (1) The NewHandPD (Pereira et al. 2016b) and (2) Mobile Device Voice Recordings at King’s College London (MDVR-KCL) (Jaeger et al. 2019). (Check Section 5: Limitations)

#### The NewHandPD dataset

**Fig. 2:**
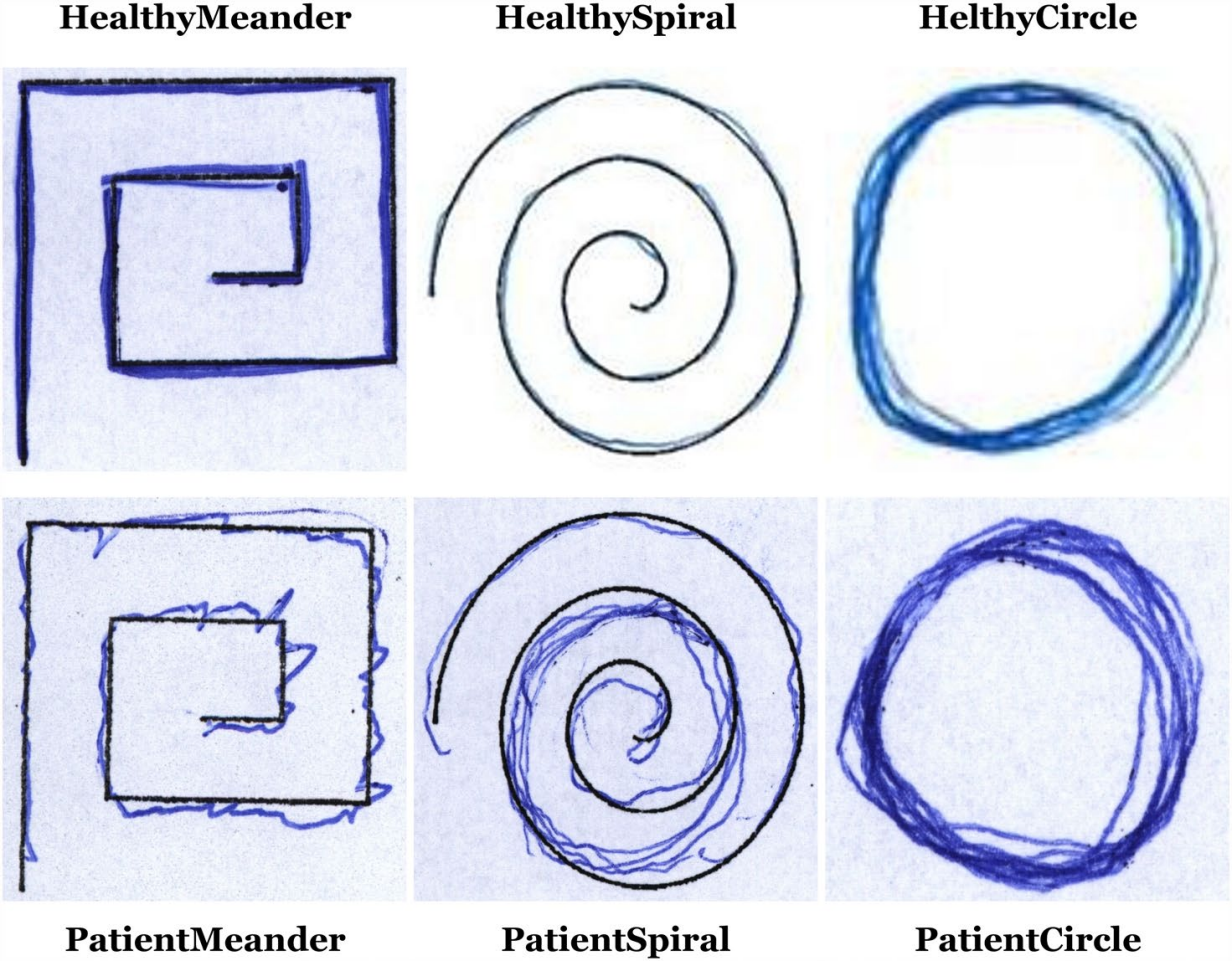
Samples from the NewHandPD dataset classes

The first dataset consists of 594 images partitioned into 6 classes, where 3 of them belong to healthy people and the remaining three belong to PD patients. They are (1) HealthyMeander (140 images), (2) HealthySpiral (140 images), (3) HelthyCircle (35 images), (4) PatientCircle (31 images), (5) PatientMeander (124 images), and (6) PatientSpiral (124 images) (Pereira et al. 2016b). Samples from it are shown in Fig. 2.

#### The MDVR-KCL dataset

The MDVR-KCL dataset consists of “SpontaneousDialogue” and “ReadText” voice records where each of them consists of 2 classes (i.e., PD for sick and HC for healthy people). The number of PD and HC cases are 15 and 21 respectively in the “SpontaneousDialogue” category and 16 and 21 respectively in the “ReadText” category (Jaeger et al. 2019).

### Pre-processing phase

The used pre-processing approaches for the images are (1) data equalization, (2) data augmentation, and (3) scale conversion while for the voice records are (1) numerical features representation, (2) graphical representation, and (3) scale conversion.

#### Images manipulation

The equalization process is applied by finding the highest class concerning the number of records and augmenting the rest of the classes’ records randomly until they reach the highest number. Data augmentation techniques are used to increase the diversity of the images, especially since the available online PD datasets are limited. The followed techniques are (1) shifting, (2) shearing, (3) zooming, (4) flipping, (5) rotation, and (6) brightness changing (Perez and Wang 2017). The augmentation is used in two locations in the current study. The first location is used before the learning process to equalize the number of records in each class. The second location is applied during the optimization and learning process with different ranges. The scale conversion includes four used techniques (1) normalization $\left( \frac{in}{255}\right)$ (Kumar and Verma 2010), (2) min-max scaling $\left( \frac{in-min(in)}{\left( max(in)-min(in)\right) }\right)$(Fulkerson and Wolfe 1962), (3) standard scaling $\left( \frac{in-\mu }{\sigma }\right)$ (Fulkerson and Wolfe 1962), and (4) max-abs scaling $\left( \frac{in}{max(|in|)}\right)$ where *in* is the input image, $\mu$ is the image mean value, and $\sigma$ is the image standard deviation value.

#### Voice records manipulation

The voice records can be processed numerically and graphically using ML or DL approaches. 16 voice feature extractions techniques are used (1) Mel-frequency Cepstral Coefficients (MFCC) using the Slaney and HTK methods (Sigurdsson et al. 2006), (2) Mel-spectrogram (Kaneko et al. 2020), (3) chroma-based techniques (chroma-only, Short-time Fourier Transform (STFT) Griffin and Lim 1984), Constant-Q Chromagram (CQT) (Liu and Xie 2012), and Chroma Energy Normalized (CENS) Kattel et al. 2019)), (4) spectral-based techniques (contrast, flatness, centroid, bandwidth, and roll-off frequency) (Bou-Ghazale and Hansen 1994), (5) Zero-crossing Rate (ZCR) (Inbar et al. 1986), (6) Tonnetz techniques (normal and harmonic) 2022 2022), and (7) Root Mean Square Error (RMSE) (Chai and Draxler 2014). The voice records are represented graphically using 5 techniques (1) spectrogram, (2) Mel-spectrogram, (3) STFT, and (4) MFCC using the Slaney and HTK methods. The STFT split the signal into time windows and runs the Fourier transform on each window to get the same information (Alsberg et al. 1997). The spectrogram is the frequency change over time. The Mel-spectrogram is the acoustic time-frequency representation. The MFCC describes the overall shape of a spectral envelope (Terasawa et al. 2012). The spectral-contrast describes the differences between the peaks and valleys in the spectrum. The Tonnetz is the tonal centroid features.

**Fig. 3:**
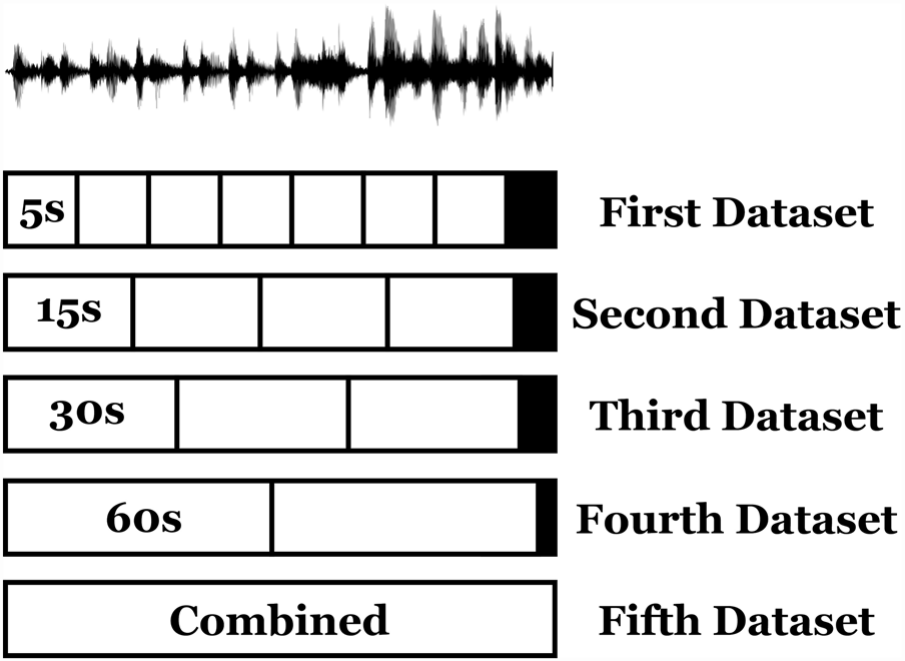
Presentation of the proposed voice records segmentation approach

**How the voice segmentation is applied (one of the study contributions)?** The input voice record is read, assuming it is a 95-seconds duration as an example. For a pre-defined segmentation duration, assuming 10 seconds, the voice is cut into 9 segments where each segment is 10-seconds in duration. The remaining 5 seconds are neglected as they are lower than the segmentation duration. This process is run again for another segmentation duration. In the current study, the voices are segmented using 5, 15, 30, and 60 segmentation durations. Also, the output numerical features and graphs from all of them are combined. Hence, the authors generated 5 numerical and graphical datasets from records. Figure 3 shows the proposed voice segmentation approach graphically.

#### Current study followed configurations

In the current study, the used pre-defined ranges in the first data augmentation location are $15\%$ shifting in the width (and height), $15\%$ shearing, $15\%$ zooming, horizontal (and vertical) flipping, $25 ^\circ$ rotation, and brightness changing with limits of [0.8, 1.2]. For the NewHandPD dataset, the number of total images after equalization is 840 (i.e., 140 images in each class with a size of (100, 100, 3)). For the MDVR-KCL dataset, the “SpontaneousDialogue” and “ReadText” voice records are combined and 281 features are extracted. Each voice record is cut into segments with different time durations (e.g., 10 seconds).

**Fig. 4:**
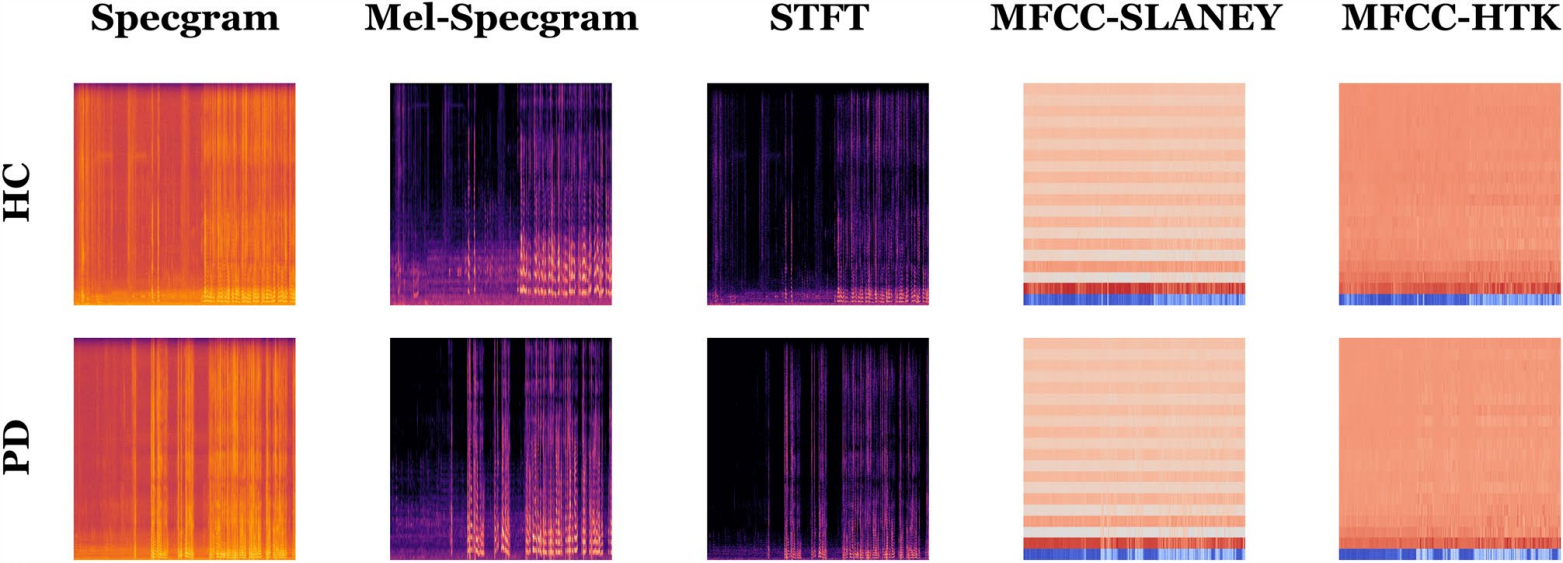
Sample graphs for each technique for the 60-second-segment-duration

Table 2 shows the number of extracted features using each mentioned technique. Table 3 shows the number of extracted graphs using each segment duration in seconds. Figure 4 shows sample graphs for each technique for the 60-second-segment-duration and they are extracted in the 480 DPI resolution.

**Table 2:** Summarization of the number of extracted numerical features for the MDVR-KCL dataset

Category	Technique	No. features
MFCC	Slaney	40
	HTK	40
Mel-Spectrogram		128
Chroma-based	Chroma-only	12
	STFT	12
	CQT	12
	CENS	12
RMSE		1
Spectral-based	Contrast	7
	Flatness	1
	Centroid	1
	Bandwidth	1
	Roll-off Frequency	1
ZCR		1
Tonnetz	Normal	6
	Harmonic	6
Total		281

**Table 3:** Summarization of the number of extracted graphs for the MDVR-KCL dataset

Segment duration (s)	No. PD	No. HC	Total
5	310	420	730
15	258	366	624
30	126	179	305
60	57	79	136
Total	751	1044	1795

### Classification and optimization phases

ML algorithms are used to classify the numerical features for the voice records dataset. The convolutional neural network (CNN) (i.e., a DL approach) is used to classify the handwritten images dataset.

#### Machine learning algorithms

The used ML algorithms are (1) decision trees (DT), (2) support vector machines (SVM), (3) Naïve Bayes (NB), and (4) K-nearest neighbor (KNN). Each ML algorithm is put in a pipeline in the order of (1) a dataset scaler layer, (2) a variance threshold layer, and (3) the ML algorithm.

#### Pre-trained CNN models

8 pre-trained CNN models on the ImageNet dataset, using the transfer learning approach (TL), are used instead of compiling models from scratch. They are (1) ResNet50, (2) VGG16, (3) VGG19, (4) MobileNet, (5) MobileNetV2, (6) MobileNetV3Small, (7) MobileNetV3Large, and (8) InceptionResNetV2 models. Each model is concatenated with a global average pooling 2D layer, a dropout layer, and an output layer. The output activation function is set to SoftMax and the pre-trained weights’ initialization is set to ImageNet. The input shape is set to (100, 100, 3) in the colored RGB mode.

#### Hyperparameters optimization

Training the models require specifying a set of hyperparameters such as batch size and dropout. The current study suggests using the grid search (GS) (with the ML algorithms) and injecting the Aquila Optimizer (AO) metaheuristic optimizer (with the CNN models) to find the best combination that will lead to the highest performance metric.

AO depends on four hunting mechanisms (1) high soar with vertical stoop in which the Aquila explores the search space (Equation 1), (2) contour flight with short glide attack in which surrounds the target (Equation 2), (3) a low flight with a slow descent attack in which the Aquila performs a vertical attack (Equation 3), and (4) walking and grab a prey in which the Aquila attacks the target (Eq. 4) Abualigah et al. 2021).

1 $$\begin{aligned}&X(t+1) = X_{best}(t)\times (1-\frac{t}{T})+(X_m(t)-X_{best}(t)\times rand) \end{aligned}$$

2 $$\begin{aligned}&X(t+1) = X_{best}(t)\times Levy(D) + X_R(t) + (y - x) \times rand \end{aligned}$$

3 $$\begin{aligned}&\begin{aligned} X(t+1) = (X_{best}(t)-X_M(t))\times \alpha -rand+ \\ ((UB-LB)\times rand + LB)\times \gamma \end{aligned} \end{aligned}$$

4 $$\begin{aligned}&\begin{aligned} X(t+1) = QF\times X_{best}(t)-(G_1\times X(t)\times rand) - \\ G_2\times Levy(D)+rand\times G_1 \end{aligned} \end{aligned}$$

where $X(t+1)$ is the solution of the next iteration $t + 1$, *X*(*t*) is the solution of the current iteration *t*, *T* is the number of iterations, $X_{best}$ is the best-obtained solution, $X_m$ is the mean location of the current solutions, *D* is the dimension value, *Levy*(*D*) is the levy flight distribution function, *y* (and *x*) are used to present the spiral shape in the search domain, *rand* is a random uniform value, *UB* is the upper bound, *LB* is the lower bound, $\alpha$ (and $\gamma$) are the exploitation adjustment parameters, $G_1$ is a notation of the various motions of the AO, $G_2$ is a decreasing value from 2 to 0, and *QF* is the quality function used to equilibrium the search techniques.

The word “generic” means that the framework can accept and handle any metaheuristic optimizer and it is not restricted by the specified optimizers in the current study.

#### Current study followed configurations

The AO population in the current study populates 10 solutions where each solution’s dimension equals 14. The reason behind this number is that each column in the solution is mapped to a specific hyperparameter randomly. These are the target hyperparameters to get optimized.

They are (1) training loss function, (2) training batch size, (3) dropout ratio, (4) TL learning ratio, (5) parameters (i.e., weights) optimizer, (6) augmentation rotation range, (7) augmentation width shift range, (8) augmentation height shift range, (9) augmentation shear range, (10) augmentation zoom range, (11) augmentation horizontal flipping, (12) augmentation vertical flipping, and (13) augmentation brightness change (“from” and “to” ranges).

In the grid search, the hyperparameters are (1) “nNeighbors” is the number of neighbors to use, (2) “leafSize” is the leaf size passed to the tree, (3) “p” is the power parameter for the Minkowski metric, (4) “criterion” is the function to measure the split quality, (5) “maxDepth” is the tree maximum depth, (6) “splitter” is the strategy used to choose the split at each node, (7) “alpha” is the additive (Laplace/Lidstone) smoothing parameter, (8) “C” is the regularization parameter, (9) “kernel” is the kernel type to be used in the algorithm, (10) “degree” is the degree of the polynomial kernel function, (11) “gamma” is the kernel coefficient, and (12) “threshold” is the threshold value used by the variance threshold layer. Table 4 shows the used ranges for each hyperparameter in the current study.

**Table 4:** The ranges for each hyperparameter

Optimizer	Category	Definition	Range
AO	CNN Learning	Loss Function	Categorical Crossentropy, Categorical Hinge, KL Divergence, Poisson, Squared Hinge, and Hinge
		Batch Size	From 8 to 64 with a step of 8
		Parameters (i.e., weights) & Optimizer	Adam, Nadam, Adagrad, Adadelta, Adamax, RMSProp, SGD, Ftrl, SGD Nesterov, RMSProp Centered, Adam, and AMSGrad
	CNN Model Structure	Dropout ratio	[0.0, 0.6]
		TL learning ratio	From 0 to 100 with a step of 1
	CNN Data Augmentation	Rotation Range	From 0 to 45 with a step of 1
		Width Shift Range	[0, 0.25]
		Height Shift Range	
		Shear Range	
		Zoom Range	
		Horizontal Flipping	[True, False]
		Vertical Flipping	
		Brightness Change (From)	[0.5, 2.0]
		Brightness Change (To)	
GS	KNN	nNeighbors	[1, 2, 3, 5, 7, 10]
		leafSize	[1, 5, 10, 15]
		p	[1, 2]
	SVM	degree	[1, 2, 3, 4, 5]
		C	[0.1, 1, 10, 100, 1000]
		gamma	[1, 0.1, 0.01, 0.001, 0.0001]
		kernel	[Linear, Poly, RBF, Sigmoid, Precomputed]
	DT	criterion	[Gini, Entropy]
		splitter	[Best, Random]
		maxDepth	From 3 to 14 with a step of 1
	NB	alpha	[0, 0.1, 0.5, 1.0, 1.5, 2, 3, 5, 10]
	Variance Threshold	threshold	[0, 0.001, 0.005, 0.01, 0.05, 0.1, 0.5]

### Performance metrics

The used performance metrics with the ML algorithms are the accuracy, F1-score, precision, recall (i.e., sensitivity), and AUC; while the used ones with the CNN learning and optimization are loss, accuracy, F1-score, precision, recall (i.e., sensitivity), specificity, AUC, IOU coefficient, and Dice coefficient.

### Framework pseudocode

The used framework pseudocode is shown in Algorithm 1. It summarizes the discussed learning and optimization phases in the suggested framework abstractly. Comments are added for illustration purposes.
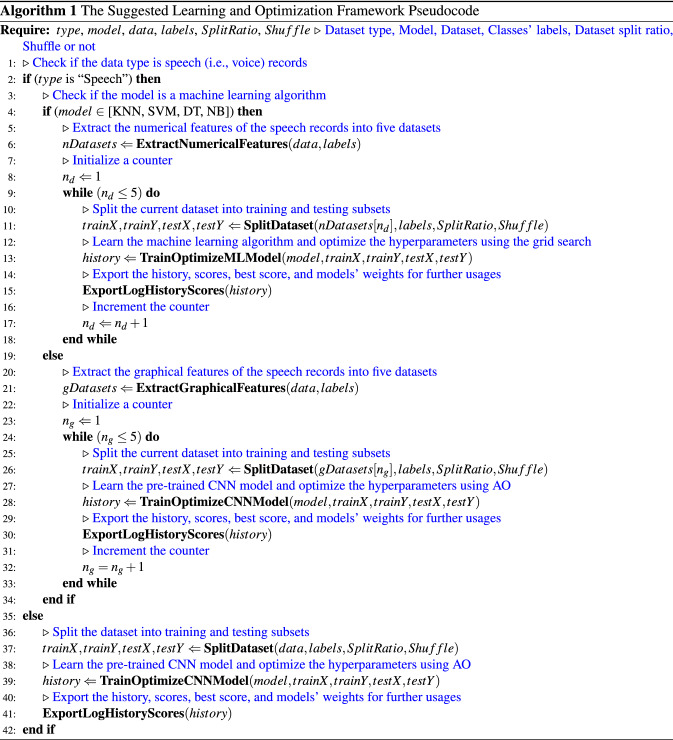


### PD patient diagnosis

After completing the framework discussion, the question is **“how can the patient perform a diagnosis?”**. In the suggested generic framework, the patient can apply two tests (1) handwriting test and (2) speech test. In the first test, the patient should draw three graphs while in the second test, the patient should read certain words (or sentences).

**Fig. 5:**
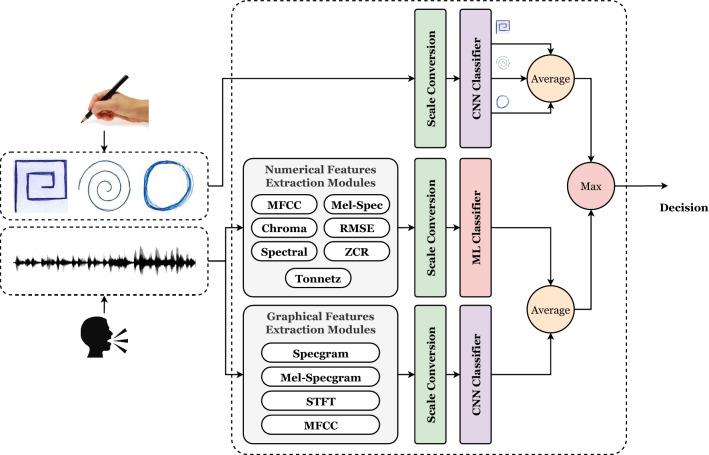
Parkinson disease (PD) patient diagnosis

The system accepts the drawn three figures as inputs, converts the scale, applies classification of each type, and takes the average of them. The system also accepts the speech record, extract the numerical and graphical features of them, convert the scale, apply classification of each type, and take the average of them. The final decision to the patient is the maximum between the two tests. It is worth mentioning that, the maximum can be changed to the average. This is summarized graphically in Fig. 5.

## Experimental results and discussion

The experiments are divided into two categories (1) experiments related to the extracted numerical features and (2) experiments related to the images and extracted graphs.

### Environment and configurations

Generally, Python is the used programming language. The learning and optimization environments are Google Colab (with its GPU) and Toshiba Qosmio X70-A with 32 GB RAM and Intel Core i7 Processor (Balaha and Saafan 2021). The NewHandPD (6 classes) and MDVR-KCL (2 classes) are the used datasets. The dataset split ratio is set to 85% (training and validation) and 15% (testing). Dataset shuffling is applied. The images (i.e., graphs) are resized to (100, 100, 3) in RGB. The train and test subsets are different so that there is no data leakage.

### First category experiments

The current subsection presents and discusses the experiments related to the extracted 281 numerical features using the mentioned ML algorithms (i.e., DT, SVM, NB, and KNN). For each ML algorithm, five experiments are applied on the 5, 15, 30, 60, and mixed durations. The algorithms are optimized using the grid search for 10 cross-validation runs, to find the best combinations with the highest metrics. The metrics (i.e., accuracy, precision, recall, F1, and AUC) are captured and reported in Table 5. It reports the best metrics 99.94%, 100%, 100%, 99.93%, and 99.95% for accuracy, precision, recall, F1, and AUC respectively. It shows that the NB algorithm reports the worst metrics. It highlights that the suggested contribution, by combining the features, reports better metrics than the individual uncombined features. The confusion matrices are presented in Table 6.

**Table 5:** Summary of the ML numerical experiments (i.e., first category experiments)

Duration (s)	Algorithm	Accuracy	Precision	Recall	F1	AUC	Scaler	Variance threshold	Best classifiers parameters
5	KNN	98.36%	97.76%	98.39%	98.07%	98.36%	Min Max	0.01	leafSize = 1, nNeighbors = 1, and p = 1
	DT	83.42%	89.54%	69.03%	77.96%	81.54%	Normalizer	0	criterion = entropy and maxDepth = 5
	NB	57.53%	0%	0%	0%	50.00%	Normalizer	0	alpha = 0
	SVM	98.90%	98.71%	98.71%	98.71%	98.88%	Min Max	0.01	C = 0.1, degree = 5, gamma = 1, and kernel = poly
15	KNN	99.04%	98.84%	98.84%	98.84%	99.01%	Max Abs	0	leafSize = 1 and nNeighbors = 1
	DT	96.63%	96.47%	95.35%	95.91%	96.44%	Normalizer	0	criterion = entropy and maxDepth = 13
	NB	58.65%	0%	0%	0%	50.00%	Normalizer	0	alpha = 0
	SVM	99.04%	98.46%	99.22%	98.84%	99.07%	Min Max	0.01	C = 100, degree = 1, and gamma = 0.1
30	KNN	98.03%	100%	95.24%	97.56%	97.62%	Max Abs	0.05	leafSize = 1, nNeighbors = 1, and p = 1
	DT	97.38%	99.17%	94.44%	96.75%	96.94%	Standardization	0.10	maxDepth = 10 and splitter = random
	NB	58.69%	0%	0%	0%	50.00%	Normalizer	0	alpha = 0
	SVM	98.03%	100%	95.24%	97.56%	97.62%	Max Abs	0.05	C = 0.1, gamma = 1, and kernel = poly
60	KNN	98.53%	100%	96.49%	98.21%	98.25%	Max Abs	0.01	leafSize = 1, nNeighbors = 1, and p = 1
	DT	83.82%	92.68%	66.67%	77.55%	81.43%	Normalizer	0	maxDepth = 3 and splitter = random
	NB	58.09%	0%	0%	0%	50.00%	Normalizer	0	alpha = 0
	SVM	94.85%	96.30%	91.23%	93.69%	94.35%	Min Max	0	C = 1, degree = 1, and gamma = 0.1
Combined	KNN	99.94%	100%	99.87%	99.93%	99.93%	Max Abs	0	leafSize = 1 and nNeighbors = 1
	DT	97.38%	96.56%	97.20%	96.88%	97.36%	Standardization	0.50	criterion = entropy and maxDepth = 14
	NB	58.16%	0%	0%	0%	50.00%	Normalizer	0	alpha = 0
	SVM	99.94%	99.87%	100%	99.93%	99.95%	Max Abs	0	C = 100, degree = 1, and gamma = 0.1

**Table 6:**
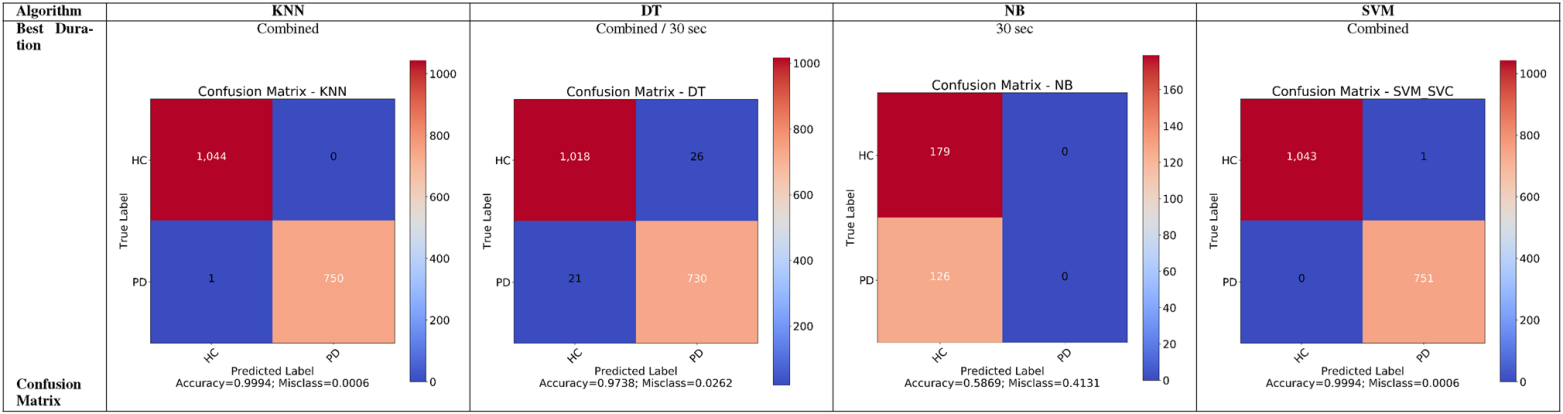
Summary of the confusion matices (i.e., first category experiments)

### Second category experiments

The current subsection presents and discusses the experiments related to the images and extracted graphical features using the mentioned pre-trained CNN models (i.e., ResNet50, VGG16, VGG19, MobileNet, MobileNetV2, MobileNetV3Small, MobileNetV3Large, and InceptionResNetV2) and AO meta-heuristic optimizer. The number of epochs is set to 5. The number of AO iterations and population size are set to 25 and 10 respectively, and hence 250 records are reported. The captured metrics are the loss, accuracy, F1, precision, recall, specificity, AUC, IOU coef., and Dice coef. as mentioned.

#### The NewHandPD experiments

**Fig. 6:**
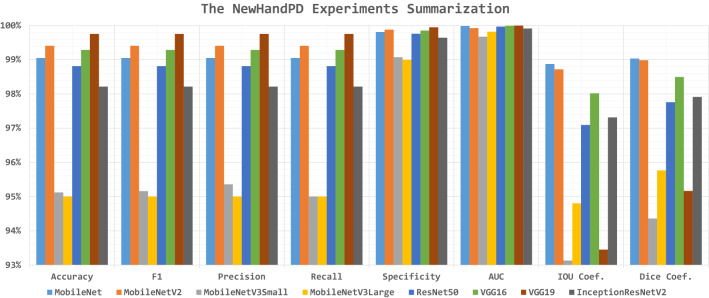
The NewHandPD experiments summarization

The top-1 record is reported concerning the testing accuracy for each pre-trained CNN model in Table 7. It shows that neglecting the horizontal and vertical flipping is preferable by six and five models respectively. The metrics results are above 93% while the best metrics are 0.029, 99.75%, 99.75%, 99.75%, 99.75%, 99.95%, 100%, 99.75%, 98.87%, and 99.04% for the loss, accuracy, F1, precision, recall, specificity, AUC, IOU coef., and Dice coef. respectively. The KL divergence loss function and SGD Nesterov (and SGD) weights optimizers are the suggested hyperparameters by the experiments. The results are graphically summarized in Fig. 6 and the correlations are reported in Table 8.

**Table 7:** The top-1 record concerning the accuracy for the pre-trained models for NewHandPD

#	MobileNet	MobileNetV2	MobileNetV3Small	MobileNetV3Large	ResNet50	VGG16	VGG19	InceptionResNetV2
Loss function	Categorical crossentropy	Categorical crossentropy	KL divergence	KL divergence	KL divergence	KL divergence	Poisson	Categorical crossentropy
Batch size	24	8	8	24	56	40	48	56
Dropout ratio	0.42	0	0.37	0.20	0.41	0.26	0.33	0.35
TL learn ratio	89%	0%	84%	55%	22%	57	67	58
Weights optimizer	SGD Nesterov	Adam	Adagrad	SGD Nesterov	Adagrad	Adagrad	SGD	SGD Nesterov
Rotation range	$29^\circ$	$0^\circ$	$39^\circ$	$7^\circ$	$4^\circ$	$36^\circ$	$31^\circ$	$32^\circ$
Width shift range	0.24	0	0.21	0.07	0.20	0.22	0.15	0.22
Height shift range	0.24	0	0.05	0.02	0.05	0.03	0.2	0.06
Shear range	0.02	0	0.21	0.23	0.19	0.15	0.15	0.13
Zoom range	0.12	0	0.22	0.25	0.01	0.19	0.22	0.20
Horizontal flip	$\checkmark$	$\checkmark$	$\times$	$\times$	$\times$	$\times$	$\times$	$\times$
Vertical flip	$\times$	$\checkmark$	$\times$	$\times$	$\checkmark$	$\checkmark$	$\times$	$\times$
Brightness range (low)	1.24	0.5	0.92	1.29	1.01	1.34	1.32	0.56
Brightness range (high)	1.28	0.5	1.08	1.52	1.67	1.76	1.46	1.3
Loss	0.038	0.032	0.152	0.107	0.049	0.029	0.180	0.049
Accuracy	99.05%	99.40%	95.12%	95.00%	98.81%	99.29%	99.75%	98.21%
F1-Score	99.05%	99.40%	95.16%	95.00%	98.81%	99.29%	99.75%	98.21%
Precision	99.05%	99.40%	95.36%	95.00%	98.81%	99.29%	99.75%	98.21%
Recall	99.05%	99.40%	95.00%	95.00%	98.81%	99.29%	99.75%	98.21%
Specificity	99.81%	99.88%	99.07%	99.00%	99.76%	99.86%	99.95%	99.64%
AUC	99.98%	99.92%	99.67%	99.82%	99.97%	99.99%	100%	99.91%
IOU coefficient	98.87%	98.72%	93.13%	94.80%	97.09%	98.02%	93.45%	97.31%
Dice coefficient	99.04%	98.99%	94.36%	95.77%	97.76%	98.50%	95.17%	97.92%

**Table 8:** The best hyperparameters correlations for the NewHandPD experiments

	Batch size	Dropout	TL learn ratio	Rotation range	Width shift range	Height shift range	Shear range	Zoom range	Horizontal flip	Vertical flip	Brightness range (low)	Brightness range (high)
Batch size	1.000											
Dropout	0.488	1.000										
TL learn ratio	− 0.037	0.663	1.000									
Rotation range	0.112	0.543	0.817	1.000								
Width shift range	0.456	0.898	0.661	0.728	1.000							
Height shift range	0.176	0.562	0.608	0.408	0.431	1.000						
Shear range	0.271	0.342	0.231	0.142	0.203	− 0.308	1.000					
Zoom range	0.047	0.251	0.731	0.646	0.259	0.144	0.558	1.000				
Horizontal flip	− 0.530	− 0.365	− 0.196	− 0.303	− 0.315	0.271	− 0.917	− 0.576	1.000			
Vertical flip	0.070	− 0.411	− 0.766	− 0.467	− 0.230	− 0.511	− 0.213	− 0.717	0.149	1.000		
Brightness range (low)	0.149	0.365	0.512	0.266	0.293	0.443	0.371	0.446	− 0.277	− 0.177	1.000	
Brightness range (high)	0.686	0.589	0.333	0.264	0.569	0.159	0.616	0.385	− 0.669	− 0.023	0.724	1.000

#### The MDVR-KCL experiments

**Fig. 7:**
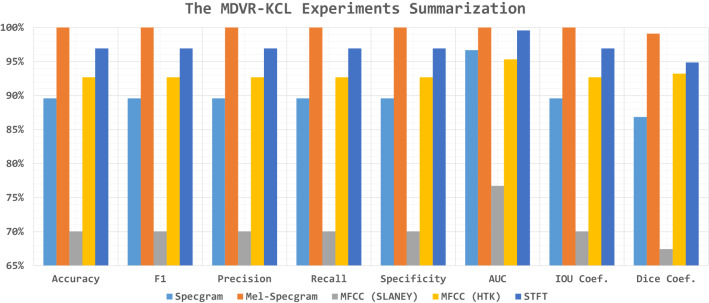
The MDVR-KCL experiments summarization

The top-1 record using VGG19 is reported concerning the testing accuracy for each combined dataset (i.e., STFT, MFCC HTK, MFCC Slaney, Specgram, and Mel-Specgram) in Table 9. The reason behind depending on the VGG19, it reported the best metrics in Table 7. It shows that neglecting the horizontal and vertical flipping is preferable by five and three models respectively. The best metrics are 0.090, 100%, 100%, 100%, 100%, 100%, 100%, 100%, 100%, and 99.09% for the loss, accuracy, F1, precision, recall, specificity, AUC, IOU coef., and Dice coef. respectively. The Poisson loss function and Adagrad (and SGD) weights optimizers are the suggested hyperparameters by the experiments. The results are graphically summarized in Fig. 7 and the correlations are reported in Table 10.

**Table 9:** The top-1 record concerning the accuracy using VGG19 for MDVR-KCL

#	Specgram	Mel-Specgram	MFCC (SLANEY)	MFCC (HTK)	STFT
Loss Function	Poisson	Poisson	Poisson	Squared Hinge	KL Divergence
Batch Size	48	40	16	48	40
Dropout Ratio	0.06	0.41	0.43	0.19	0.31
TL Learn Ratio	86	67	13	59	61
Weights Optimizer	Adagrad	SGD	RMSProp Centered	Adagrad	SGD
Rotation Range	29	29	41	41	35
Width Shift Range	0.14	0.2	0	0.09	0.16
Height Shift Range	0.09	0.14	0.15	0.05	0.2
Shear Range	0.01	0.14	0.22	0.13	0.14
Zoom Range	0.13	0.13	0.01	0.13	0.17
Horizontal Flip	$\times$	$\times$	$\times$	$\times$	$\times$
Vertical Flip	$\checkmark$	$\times$	$\times$	$\checkmark$	$\times$
Brightness Range (Low)	0.72	1.33	0.55	0.65	1.38
Brightness Range (High)	1.32	1.65	1.16	1.33	1.41
Loss	0.619	0.505	0.791	0.654	0.090
Accuracy	89.58%	100%	70.03%	92.68%	96.93%
F1-Score	89.58%	100%	70.03%	92.68%	96.93%
Precision	89.58%	100%	70.03%	92.68%	96.93%
Recall	89.58%	100%	70.03%	92.68%	96.93%
Specificity	89.58%	100%	70.03%	92.68%	96.93%
AUC	96.69%	100%	76.72%	95.33%	99.55%
IOU Coefficient	89.58%	100%	70.03%	92.68%	96.93%
Dice Coefficient	86.85%	99.09%	67.41%	93.22%	94.85%

**Table 10:** The best hyperparameters correlations for the MDVR-KCL experiments

	Batch size	Dropout	TL learn ratio	Rotation range	Width shift range	Height shift range	Shear range	Zoom range	Horizontal flip	Vertical flip	Brightness range (low)	Brightness range (high)
Batch size	1.000											
Dropout	− 0.743	1.000										
TL learn ratio	0.923	− 0.701	1.000									
Rotation range	− 0.456	0.241	− 0.752	1.000								
Width shift range	0.688	− 0.169	0.816	− 0.812	1.000							
Height shift range	− 0.485	0.624	− 0.292	− 0.130	0.155	1.000						
Shear range	− 0.792	0.886	− 0.900	0.664	− 0.519	0.423	1.000					
Zoom range	0.863	− 0.445	0.836	− 0.495	0.848	0.006	− 0.578	1.000				
Horizontal flip	N/A	N/A	N/A	N/A	N/A	N/A	N/A	N/A	1.000			
Vertical flip	0.667	− 0.910	0.519	0.000	− 0.036	− 0.886	− 0.703	0.241	N/A	1.000		
Brightness range (low)	0.248	0.324	0.372	− 0.536	0.817	0.640	− 0.005	0.657	N/A	− 0.555	1.000	
Brightness range (high)	0.462	0.186	0.554	− 0.671	0.898	0.162	− 0.179	0.624	N/A	− 0.250	0.816	1.000

### Time complexity remarks

The major objective of the current study was to build a framework for the PD using speech and handwritten datasets. The target was to achieve high performance metrics as reported in the results. The learning and processing time was high and hence was not reported exactly in the study. However, approximate times can be calculated. It is worth noting that the time depended mainly on the working environment. The current study worked on two environments as mentioned in Sect. 4.1. For the ML model, the second environment is used while the first environment is used with the CNN models.

For the first category experiments, the GS and 10-folds CV are used. Assuming that, each ML model takes one second approximately. Table 11 shows the approximate time for each ML model. The total approximate time for the ML models for a single dataset is 7,300 seconds (i.e., 121 minutes or 2 hours). We have 5 datasets as shown in Tables 3 and 5. Hence, we need 10 hours approximately to launch them. Of course, they are approximate times and the ML models may take longer than this.

By applying the same concept with the pretrained models but with the assumption that each model takes 1 minutes due to the model complexity. There are 14 hyperparameters to optimize using the AO. The number of iterations is set to 10, the population size is set to 25, and the number of epochs is set to 5. Hence, there are $10 \times 25 \times 5 = 1,250$ runs for each model to complete. The approximate time is 1,250 minutes (i.e., 20 hours) for a single model. We have 8 pretrained models in Table 7 and 5 datasets in Table 9. Hence, there are 14 experiments. The total approximate time can be 291 hours (i.e., 12 days).

**Table 11:** Approximate times for each ML model

Model	Hyperparameters #	Total configurations #	With 10 folds	Approximate time (s)
KNN	3	$6 \times 4 \times 2 = 48$	480	480
SVM	4	$5 \times 5 \times 5 \times 5 = 625$	6,250	6,250
DT	3	$2 \times 2 \times 12 = 48$	480	480
NB	1	9	90	90
				7300

### Related studies comparisons

Table 12 shows a comparison between the suggested approach and related studies concerning the same used datasets.

**Table 12:** Related studies comparisons

References	Best accuracy	Other metrics
Pereira et al. 2015)	78.9%	–
Pereira et al. 2016b)	80.19%	–
Pereira et al. 2016a)	90.39%	–
Pereira et al. 2018)	95%	–
Senatore et al. 2019)	72.36%	–
Impedovo 2019)	98.44%	–
Naseer et al. 2020)	98.28%	85.98% precision, 67.57% sensitivity, and 76.37% specificity
Kamran et al. 2021)	99.75% (CNN-TL)	–
Sakar et al. 2013)	77.5%	–
Caliskan et al. 2017)	86.09%	58.27% sensitivity abd 95.39% specificity
Goyal et al. 2021)	99.37%	–
Tuncer and Dogan 2019)	97.62% by 1NN	97.61% F1
Zahid et al. 2020)	99.7%	–
Proposed approach	99.94% (ML)	Table 5
Proposed approach	99.75% (NewHandPD)	Table 7
Proposed approach	100% (MDVR-KCL)	Table 9

## Limitations

The major limitation of the current study is the dataset as the PD public and available datasets are limited and there is no dataset that contains handwriting and voice data for the same patient. In the future, data from PD patients can be collected as well to further validate the effect of diversity based on the suggested approach.

## Conclusions and future work

Parkinson’s disease is a progressive and chronic disorder that harms the life of the patients. Scientists are still trying to find a suitable treatment for the disease. The main problem of PD is that patients are correctly diagnosed in the late stages. Therefore, a massive effort is done to diagnose PD in its early stages to use the proper medication to control the symptoms as possible. The challenge is that the symptoms of PD are similar to other diseases. In this paper, the authors proposed a comprehensive generic framework for the early diagnosis of PD using a combination of disorders in handwritten and (or) speech signals. For handwriting disorders, 8 pre-trained deep CNNs via TL, namely ResNet50, VGG16, VGG19, MobileNet, MobileNetV2, MobileNetV3Small, MobileNetV3Large, and InceptionResNetV2, are used to diagnose PD using the handwritten spirals drawn by PD patients. To maintain an enhanced performance, the authors used a metaheuristic optimizer, namely the Aquila optimizer, to optimize the hyperparameters in the different CNN structures to achieve the best structure. For voice signals, both numerical and graphical features are extracted. Numerical features are extracted using 16 feature extraction algorithms, namely: (1) MFCC using the Slaney and HTK methods, (2) Mel-spectrogram, (3) chroma-based techniques (chroma-only, STFT, CQT, and CENS), (4) spectral-based techniques (contrast, flatness, centroid, bandwidth, and roll-off frequency), (5) ZCR, (6) Tonnetz techniques (normal and harmonic), and (7) RMSE. These features are used in 4 machine learning (ML) algorithms, namely Decision Tree (DT), Support Vector Machine (SVM), Naïve Bayes (NB), and K-Nearest Neighbor (KNN). The grid search algorithm is applied to optimize the parameters of the different ML algorithms. Graphical features are extracted using 5 techniques, namely (1) spectrogram, (2) Melspectrogram, (3) STFT, and (4) MFCC using the Slaney and HTK methods. These features are applied to the different pretrained CNN structures. One of the major contributions of the current work is proposing a new feature extraction algorithm. The idea of the proposed algorithm is to use a dataset of voice segments divided by different durations to guarantee a variety in the features. For the NewHandPD dataset, the best-reported metrics are 99.75% using the VGG19 structure. For the MDVR-KCL dataset, the best-reported metrics are 99.94% using the KNN and SVM ML algorithms and the combined numerical features; and 100% using the combined the mel-specgram graphical features and VGG19 structure. These results are better than other state-of-the-art researches.

### Future work

The proposed framework and suggested approach can be improved by adding other biomarkers and datasets including UPDRS scores for the classification of PD based on severity. Other deep learning classifiers such as recurrent neural networks (RNN) can be used for frequency-time data. The authors plan to apply the proposed framework to other diseases such as Alzheimer’s and heart diseases. Instead of pre-trained models, we also plan to build a CNN model from scratch for the framework. We also plan to use CNN in the pre-processing phase due to its powerful features.

## Appendix A: Table of abbreviations

The table of abbreviations is shown in Table 13.

**Table 13:** Table of abbreviations

Abbreviation	Definition
PD	Parkinson’s disease
CNN	Convolutional neural network
TL	Transfer learning
ML	Machine learning
AO	Aquila optimizer
GS	Grid search
DL	Deep learning
DT	Decision tree
SVM	Support vector machine
NB	Naive Bayes
KNN	K-Nearest neighbor
PSD	Parkinson speech dataset
OPD	Oxford Parkinson’s disease detection dataset
MFCC	Mel-frequency cepstral coefficients
STFT	Short-time Fourier transform
CQT	Constant-Q chromagram
CENS	Chroma energy normalized
ZCR	Zero-crossing rate
RMSE	Root mean square error
